# Dexmedetomidine versus remifentanil in nasal surgery: a systematic review and meta-analysis

**DOI:** 10.1186/s12871-024-02563-0

**Published:** 2024-05-30

**Authors:** Masoud Janipour, Shahin Bastaninejad, Alireza mohebbi, Amin Amali, Seyed Hossein Owji, Kimia Jazi, Rojan Abdollahzadeh Mirali, Reza Moshfeghinia

**Affiliations:** 1 Otolaryngology Research Centre, Department of Otolaryngology, Shiraz University of Medical Sciences, Shiraz, Iran; 2 Otorhinolaryngology Research Center, Amiralam Hospital, Tehran University of Medical Sciences, Tehran, Iran; 3 ENT and Head and Neck Research Center and Department, The Five Senses Health Institute, School of Medicine, Iran University of Medical Sciences, Tehran, Iran; 4 Otorhinolaryngology Research Center, Otorhinolaryngology Head and Neck Surgery Department, Imam Khomeni Hospital Complex, Tehran University of Medical Sciences, Tehran, Iran; 5 Student Research Committee, Shiraz University of Medical Sciences, Shiraz, Iran; 6 Student Research Committee, Faculty of Medicine, Medical University of Qom, Qom, Iran; 7 Research Center for Psychiatry and Behavior Science, Shiraz University of Medical Sciences, Shiraz, Iran

**Keywords:** Dexmedetomidine, Nasal surgery, Remifentanil, Rhinoplasty

## Abstract

**Background:**

Nasal surgeries, addressing anatomical variations for form and function, require careful anesthesia administration, including dexmedetomidine and remifentanil. This meta-analysis evaluates their safety and efficacy variations in nasal surgeries, emphasizing patient comfort and optimal outcomes.

**Methods:**

Four electronic databases (PubMed, Scopus, Web of Science, and CINAHL Complete) were searched for records in English. Studies that measure the effect of dexmedetomidine versus remifentanil on patients underwent nasal surgery were included. The Cochrane Collaboration’s tool was used to assess the quality of the included studies. A random-effect model was preferred and statistical analysis was performed by Stata software version 17.

**Results:**

Out of an initial pool of 63 articles, five studies were selected for this analysis. All of these chosen studies were Randomized Controlled Trials (RCTs). The meta-analysis involved a total of 302 participants, with 152 in the remifentanil group and 150 in the dexmedetomidine group. The analysis aimed to compare the effects of Dexmedetomidine and Remifentanil on heart rate (HR) and mean arterial pressure (MAP) during surgery. Both groups exhibited similar MAP and HR, with the exception of a slightly lower HR in the remifentanil group at the 15th minute of surgery (Standardized Mean Difference: -0.24 [-0.83, 0.34]). Furthermore, when evaluating the impact of these medications on post-surgery outcomes, including pain levels, the use of pain relief medications, patient-surgeon satisfaction, agitation scores, and recovery time, no significant differences were observed between the two medications in any of these aspects.

**Conclusion:**

In summary, the study compared Dexmedetomidine and Remifentanil in nasal surgeries anesthesia. No significant differences were found in heart rate, blood pressure, satisfaction, pain, agitation, or recovery time. The study had limitations, and future research should establish standardized protocols and consider various surgical factors.

**Supplementary Information:**

The online version contains supplementary material available at 10.1186/s12871-024-02563-0.

## Introduction

Rhinoplasty entails intricate adjustments to the complex nasal structure, involving bones, cartilage, and vasculature. Despite its apparently simple goal of reshaping the nose, the surgery is complex due to the unique anatomical variations in nasal structure, necessitating careful consideration by surgeons [1]. The procedure carries emotional, physical, and financial burdens associated with potential risks, post-operative dissatisfaction, and the recovery process. Epidemiologically, rhinoplasty is a globally prevalent cosmetic surgery, with approximately 295,000 procedures performed in the United States in 2021 [2]. Anesthesia, medication choices, and postoperative care contribute to the multifaceted nature of rhinoplasty [3, 4], underscoring the importance of skilled surgical teams, effective communication, and functional considerations for optimal outcomes. These concerns are particularly relevant to anesthesia administration [5, 6].

Administering anesthesia for nasal procedures with rich vascularity requires careful attention to balancing deep sedation, patient comfort, and prompt emergence for postoperative assessment [7]. Rhinoplasty patients are at risk of airway obstruction, which can lead to inadequate oxygenation, respiratory distress, and, in severe cases, necessitate reintubation [8]. The selection of anesthetic agents and their dosages is crucial in this scenario, tailored to meet the specific requirements of the surgical procedure and the individual patient [9].

Dexmedetomidine, an alpha-2 adrenergic agonist, offers sedation, hemodynamic stability, and analgesia in rhinoplasty. Its unique mechanism of action addresses the emergence of agitation after nasal surgery [10]. Remifentanil, a potent opioid, stands out for rapid onset and offset of analgesia, aiding intraoperative and postoperative pain control. It enhances hemodynamic stability, blocks stimuli during surgery, and facilitates faster patient recovery [11]. Dexmedetomidine and Remifentanil differ in onset and duration of action. With a slower onset, Dexmedetomidine is suitable for prolonged sedation, maintaining stable hemodynamics. It prevents emergence agitation, notably in nasal and ophthalmic surgeries. Remifentanil provides rapid pain relief but necessitates continuous administration due to its short duration. It is favored for immediate, intense pain control in surgical settings. The choice between them depends on surgery and patient characteristics, acknowledging the merits of both medications [5, 6, 9].

Research delves into the significant impact of anesthesia choices on patient comfort and recovery after rhinoplasty. One study evaluates the effectiveness of medications in managing emergence agitation following sevoflurane anesthesia, attributing their impact to their calming and pain-relieving properties [9]. Another investigation by F. Zamani focuses on reducing bleeding during rhinoplasty, demonstrating that remifentanil notably decreases mean arterial pressure and intraoperative bleeding compared to dexmedetomidine [11]. Surgeon satisfaction in an Iranian study leans towards dexmedetomidine due to its reduced bleeding outcomes [6]. Despite slight discrepancies in reported complications, the overall efficacy of medications remains consistent [5, 9, 12]. These studies underscore different scenarios for medication use, one addressing postoperative agitation and the other focusing on intraoperative bleeding control. The limited literature on this comparison underscores the significance of comprehending their distinct mechanisms and characteristics. An exploration of these drugs can yield insights into tailored approaches for post-operative care. Considering their diverse pharmacological profiles, the exploration and comparison of Dexmedetomidine and Remifentanil are essential in determining the optimal medication for adults undergoing general anesthesia.

The focus is on improving patient safety and surgical outcomes through appropriate surgical methods, anesthesia, and medications. The complexity of nasal anatomy and the need for balanced anesthesia require thorough investigation. Examining the advantages of medications aims to enhance medical practices, ensure a safer post-operative experience for patients, and benefit the art and science of the procedure. This systematic review aims to assess the effectiveness and safety of Dexmedetomidine compared to Remifentanil in nasal surgeries, with potential implications for surgeon satisfaction.

## Methods

This systematic review and meta-analysis followed the Preferred Reporting Items for Systematic Reviews and Meta-Analyses (PRISMA) guidelines 2020 [13]. The registration number in PROSPERO is CRD42023475182, and its protocol is available.

### Search strategy

Four electronic databases (PubMed, Scopus, Web of Science, and CINAHL Complete) were systematically searched for English-language records from their inception to September 29, 2023. The searches utilized keyword combinations such as “Dexmedetomidine” AND “Remifentanil” AND “Rhinoplasty” OR “Nasal surgery.” Synonyms and related terms were included in the search strategy. Detailed search strategies for each database can be found in Supplementary Material 1. Additionally, the references of the included studies were screened to identify potentially eligible articles.

### Eligibility criteria

We included the randomized controlled trials (RCTs) that assessed the effects of Dexmedetomidine versus Remifentanil on nasal surgery outcomes. Based on PICO (Population, Intervention, Comparison, and Outcomes), the following criteria are the inclusion criteria:

Population: Patients undergoing nasal surgery.

Intervention and Comparison: Dexmedetomidine and Remifentanil were administered during surgery for anesthesia, with one serving as the intervention and the other as the control.

Outcomes: The primary outcome was heart rate and mean arterial pressure during nasal surgery, and the secondary outcomes were bleeding, satisfaction levels among patients and surgeons, pain intensity, and other relevant outcomes.

Additionally, studies were excluded based on the following criteria:

- Insufficient data for calculating differences in nasal surgery outcomes between the Dexmedetomidine and Remifentanil groups.
- Duplication of studies or overlapping participant data.
- Studies falling into reviews, cohorts, case-control, cross-sectional, editorials, conference papers, case series/reports, secondary analyses, or animal experiments.
- Studies utilizing qualitative research designs.

### Study selection

Two authors, KJ and RA, conducted independent reviews of the titles and abstracts of potentially eligible studies using EndNote. For studies deemed potentially eligible, separate authors independently evaluated the full texts. Any conflicts pertaining to study design or methods, as well as the final decision on whether to include studies, were resolved through a consensus meeting chaired by RM.

### Data extraction

Two authors, KJ and RA, independently extracted information from the included articles. Any discrepancies were resolved through further discussions. The following general characteristics were collected: author names and publication years, study location, study design, sample size, ethnicity, male-to-female ratio, and the primary findings of the included studies (refer to Tables 1 and 2).

**Table 1:** General characteristics of included studies

Author	Country	Participants	Male %	Dexmedetomidine	Remifentanil
Kavalci, et al. 2013 [9]	Turkey	60	Group D: 53% male *N* = 30 Mean Age:31.47 Group R: 60% male *N* = 30 Mean Age:33	A loading dose of 1 µg/kg was given to Group D (*n* = 30) in 100 ml 0.9% saline in 10 min.	A loading dose of 1 µg/kg was given to Group R (*n* = 30) in 100 ml 0.9% saline in 10 min.
Ersoy, et al. 2023 [5]	Turkey	62	Group D: 19% male *N* = 31 Mean Age:29.1 Group R: 42% male *N* = 31 Mean Age:32.19	In group D, 1 µg/kg IV bolus of dexmedetomidine was given 10 min before induction, and it was administered at a 0.5 µg/kg/hr infusion rate throughout the surgery.	In group R, remifentanil was administered at a 0.25 µg/kg/min infusion rate with pump throughout the whole procedure without a loading dose.
Jouybar, et al. 2022 [6]	Iran	60	Group D: 10% male *N* = 30 Mean Age:28.2 Group R: 17% male *N* = 30 Mean Age: 32.1	patients received an IV infusion of 1 µg/kg dexmedetomidine over 20 min before induction of anaesthesia then 0.6 µg/kg/hr. medication from the time of induction until the end of the operation.	patients received remifentanil at an infusion rate of 0.25 µg/kg/mi with infusion pump from the time of anaesthesia induction until the end.
Polat, et al. 2015 [12]	Turkey	90	Group D: 73% male *N* = 30 Mean Age:32 Group R: 60% male *N* = 30 Mean Age:37 Group S: 66% male *N* = 30 Mean Age:36	The group D (*n* = 30), received dexmedetomidine infusion at a rate of 0.4 µg/kg.h	The group R (*n* = 30), received remifentanil infusion at a rate of 0.05 µg/kg.h
Zamani, et al. 2020 [11]	Iran	60	Group D: 23% male *N* = 30 Mean Age:23.9 Group R: 22% male *N* = 30 Mean Age:23.2	After induction of anaesthesia, the patients were intubated and placed under the ventilator then patients in each group(*n* = 30) received the intervention.	
				0.5 µg/kg/h dexmedetomidine infusion was administered.	50–100 µg/kg Remifentanil infusion was given.

**Table 2:** General characteristics of included studies

Author	Defining of groups	Method used for surgery	Main findings	Pathophysiology	Limitations	
Kavalci, et al. 2013 [9]	Age: 18–65 years, ASA I-II Class, undergoing septoplasty operation	Premedication: No premedication, Ringer’s lactate solution at a rate of 5 ml/kg/h during perioperative period was infused instead. Maintenance anaesthesia: It was achieved by 50% oxygen + 50% air and 5% sevoflurane	Remifentanil and Dexmedetomidine had similar effects on emergence agitation. Hemodynamic stability, Demographic data and VAS score was also not significantly different between both groups.	Sedative and analgesic effects of dexmedetomidine and remifentanil has been discussed.	Potential biases in patient selection and group assignment. No control groups. Not enough investigation for the changes of patients’ hemodynamic	
Ersoy, et al. 2023 [5]	Age: 18–65 years, ASA I-II class, undergoing elective rhinoplasty operation	Premedication: 0.03 mg/kg Midazolam IV Maintenance anaesthesia:2–3% sevoflurane in a half air-oxygen mixture in both groups was set.	Both groups did not differ in the anaesthesia duration or surgery, the amount of fluid administered during surgery, or the dose of IV paracetamol. Pain scores were lower in the dexmedetomidine group and less shivering was experienced.	Not mentioned.	No control groups. Potential bias from the surgical field.	
Jouybar, et al. 2022 [6]	Age: 18–45 years, ASA I-II class, undergoing rhinoplasty.	Premedication: No premedication, Anaesthesia: IV infusion of propofol (150 µg/kg/min for the first twenty minutes, then 120 µg/kg/min for the second twenty minutes, then 100 µg/kg/min for the rest of the time. (A mixture of 50% oxygen and 50% NO was received additionally.)	Surgeon satisfaction was significantly higher in Group D due to the less amount of bleeding reported. respiration and extubation was longer in the group R than in the group D. There was also an initial painlessness reported in group R which made the VAS score relatively lower than the other group.	Since the blood flow is reduced the bleeding is controlled and the surgical is more accessible for surgeon.	Possible bias for the method of Anaesthetic intervention administration.	
Polat, et al. 2015 [12]	Age:18–65 years with ASA I-II class, who underwent general anaesthesia for elective nasal surgery in which nasal packing was used on each side after surgery.	Premedication: oral midazolam 0.2 mg/kg 30 min before procedure. Maintenance anaesthesia: desflurane in 50% O2/N2O	Hemodynamics was similar in both groups during surgery. Comparing both drugs infusion, Remifentanil was superior in avoiding EA, however, Patients who received dexmedetomidine had less vomiting and pain. It was marked that without the use of a loading dose, anaesthetic maintenance with either remifentanil or dexmedetomidine infusion until extubation is more effective in preventing EA than saline.	Both of the drugs reduce agitation and provide sedation with adequate respiration and help stabilizing patient hemodynamics.	Potential biases in patient selection and groups and patients self-reported data.	
Zamani, et al. 2020 [11]	Age: 18–50 years, ASA I-II class, undergoing rhinoplasty surgery lasting 90–180 min.	Premedication: CVE of 3–5 cc/kg of crystalloid fluid. 1 mg midazolam plus 1-2 cc fentanyl was also given. Anaesthesia: 3–5 µg/kg fentanyl, 0.2 mg/kg midazolam, 0.5 µg/kg atherocurium and 2–3 mg/kg propofol.	Patients receiving remifentanil had lower BP while in the other groups individual were more bradycardia. Controlling hypotension and reducing intraoperative bleeding was more significant in Group R.	Not mentioned.	Limited sample size- No placebo- No investigation for postoperative complications.	

*Abbreviations* ASA, American Society of Anesthesiologists;

### Quality assessment

The risk of bias and the quality of the included studies are assessed using Cochrane tools [14]. The Risk of Bias-2 (ROB-2 tool), also known as the Cochrane Risk of Bias Tool for Randomized Trials [15], is structured into five domains (D) for evaluating potential bias. Study quality is assessed in three categories: high risk of bias, some concerns, and low risk of bias. The total score on the scale can be used to determine one of four quality ratings: low, some concerns, or severe risk of bias.

### Quantitative analysis

Standardized mean differences (SMDs) were employed to account for variations in outcome measurement methods across diverse studies. In our research, we utilized SMDs and a 95% confidence interval (CI). The mean and standard deviation (SD) were calculated based on the median, range, or interquartile range (IQR), following the methodology outlined by Wan et al. [16]. The Cochrane Q-test and I2 index were used to assess between-study heterogeneity. It is important to note that, for the Cochrane’s Q-test, a P-value below 0.05 was considered statistically significant, and I2 values of 0.75, 0.50, and 0.25 respectively indicated high, moderate, and low levels of heterogeneity. Furthermore, a random-effects model (restricted maximum likelihood model) was employed for meta-analysis when dealing with heterogeneous results; otherwise, we consistently used the random-effects model. Statistical analyses of the differences in outcomes between Dexmedetomidine and Remifentanil were conducted using STATA 17.0 (Stata Corporation, College Station, TX, USA). Unless stated otherwise, a P-value less than 0.05 was considered statistically significant.

## Results

### Selection of studies

**Fig. 1:**
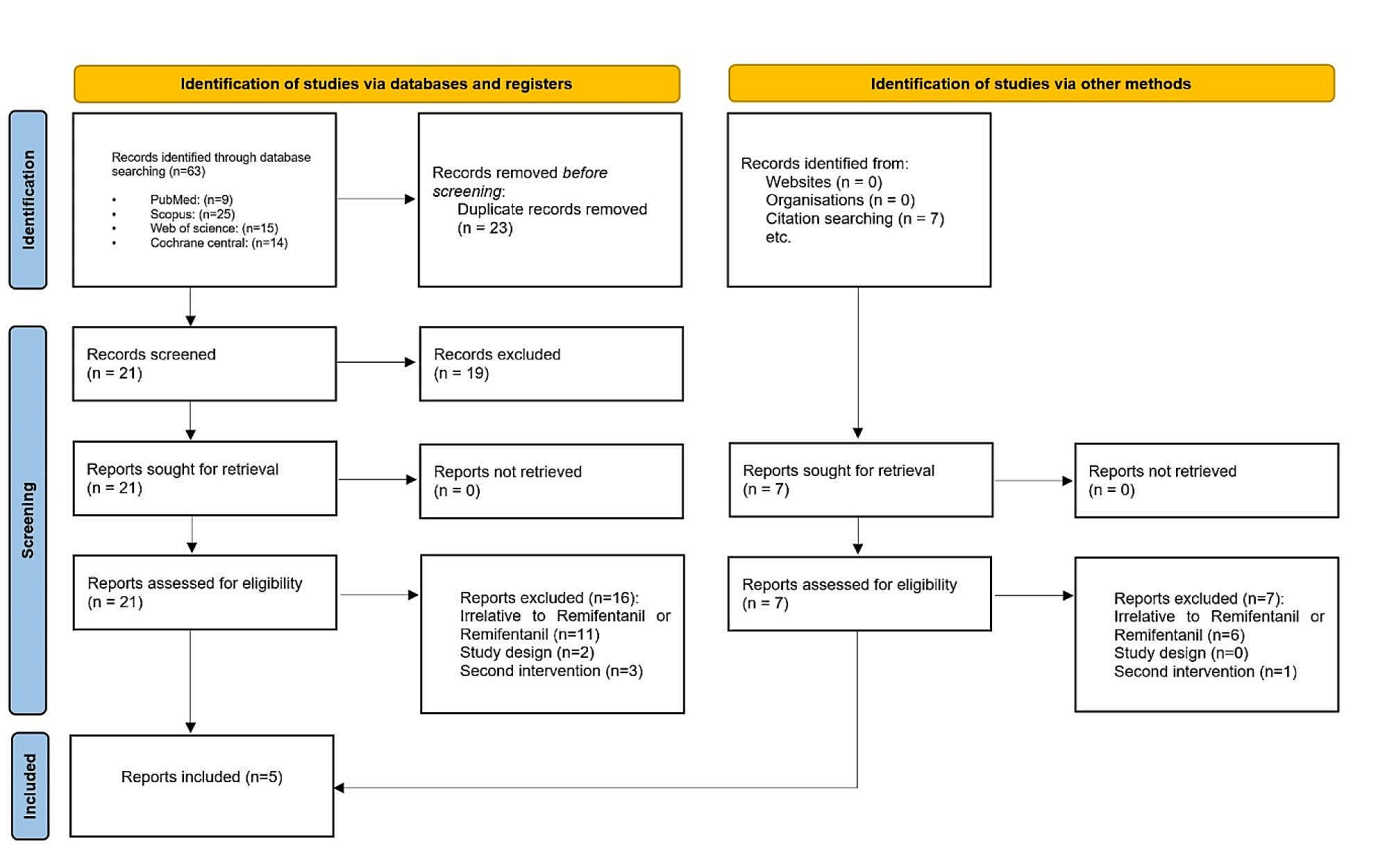
The Preferred Reporting Items for Systematic Reviews and Meta-Analysis (PRISMA) flow diagram of search results

Figure 1 illustrates the PRISMA flowchart. Initially, the search criteria generated 63 articles. After eliminating 23 duplicates using EndNote, we excluded 19 articles following title and abstract screening. Subsequently, based on the eligibility criteria, we identified 21 articles as potentially relevant to our systematic review. Following a thorough evaluation of the full texts, 16 articles were excluded, resulting in five [5, 6, 9, 11, 12] articles remaining.

### Study characteristics

The research originates from diverse countries, such as Turkey [5, 9, 12] and Iran [6, 11], and involves participants with varying demographics. Notably, although the male proportion in these groups differed, the majority were females. The use of dexmedetomidine and remifentanil also exhibited diversity in terms of loading doses and infusion rates. For example, Kavalci et al. [9] detailed Group D and Group R, where a 1 µg/kg Dexmedetomidine loading dose was administered over 10 min in 100 ml 0.9% saline to Group D, while Group R received a similar Remifentanil dosage. Ersoy et al. [5] provided Dexmedetomidine at a one µg/kg IV bolus 10 min pre-surgery in one group and a continuous Remifentanil infusion at 0.25 µg/kg/min throughout the procedure in another group without a loading dose. Jouybar et al. [6] and Polat et al. [12] from Iran and Turkey, respectively, used different concentrations of Dexmedetomidine and Remifentanil at varied infusion rates in their participant groups. Furthermore, Zamani et al. [11] from Iran focused on interventions post-anesthesia induction, administering a 0.5 µg/kg/h Dexmedetomidine infusion or a 50–100 µg/kg Remifentanil infusion (Table 1).

The studies consistently reported a relatively uniform patient age range and ASA class (I-II), with slight variations. Diverse premedication methods were employed, ranging from midazolam administration in some studies [6, 12] to no premedication in others [6, 9]. Anesthesia maintenance techniques also varied, including a combination of oxygen, air, and sevoflurane in some studies [5, 6, 12] and IV infusions of propofol or sevoflurane in an air-oxygen mixture in others [9, 11]. Certain studies found comparable effects of dexmedetomidine and remifentanil on emergence agitation, hemodynamic stability, and pain control [9, 12] while differences between the two groups were significant in studies with different interventions [6, 11]. Kavalci et al. [9] highlighted similarities in the effects of both medications during septoplasty but noted limitations in patient selection and hemodynamic changes. Ersoy et al. [5] reported lower pain scores and less shivering with dexmedetomidine during rhinoplasty, and Jouybar et al. [6] found better surgeon satisfaction with dexmedetomidine, along with differences in respiration time and initial pain compared to remifentanil in the same operation. Polat et al. [12] advocated for the use of remifentanil to prevent emergence agitation but highlighted dexmedetomidine’s benefits in reducing vomiting and pain, particularly stressing the importance of a loading dose. Zamani et al. [11] showcased the significance of remifentanil in decreasing intraoperative bleeding during rhinoplasty. While there were variations in specific findings and outcomes, the studies collectively emphasized similarities in certain effects between dexmedetomidine and remifentanil in managing emergence agitation and pain control. However, disparities were observed in areas such as shivering, surgeon satisfaction, and hemodynamic changes, highlighting nuanced differences in the impacts of these medications across various surgical contexts [5, 6, 9, 11, 12]. Despite some distinctions, most results tended to converge for both medications, with certain studies pointing to reduced bleeding and enhanced surgical accessibility with specific medications [6, 11]. Common limitations across the studies included the absence of control groups (except for the study conducted by Polat et al. [12] who incorporated a control group), potential biases in patient selection and group allocation, and reliance on self-reported data. Other limitations encompassed inadequate exploration of certain changes, such as hemodynamic alterations, and potential bias from the surgical field (Table 2).

### Risk of bias within studies

**Fig. 2:**
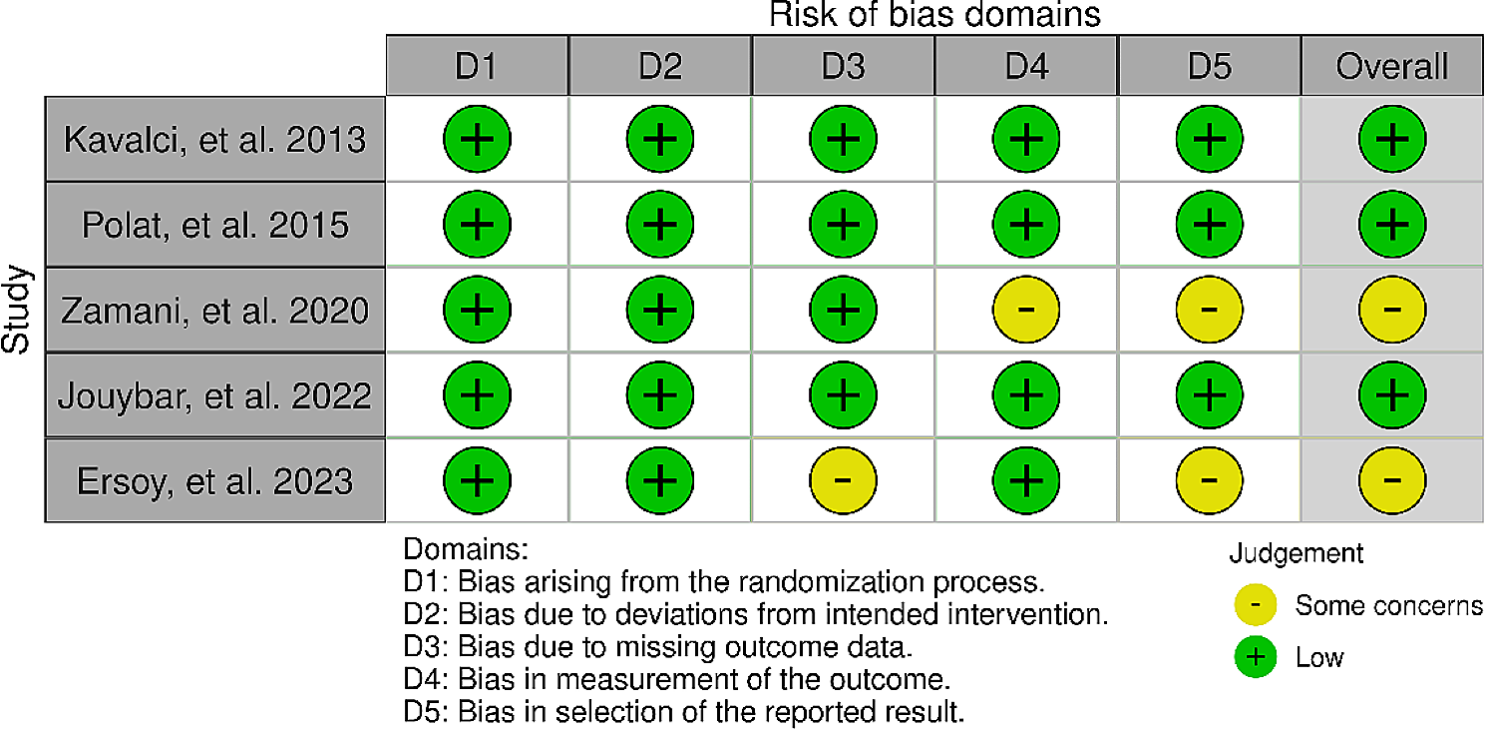
Quality assessment of the included studies

**Fig. 3:**
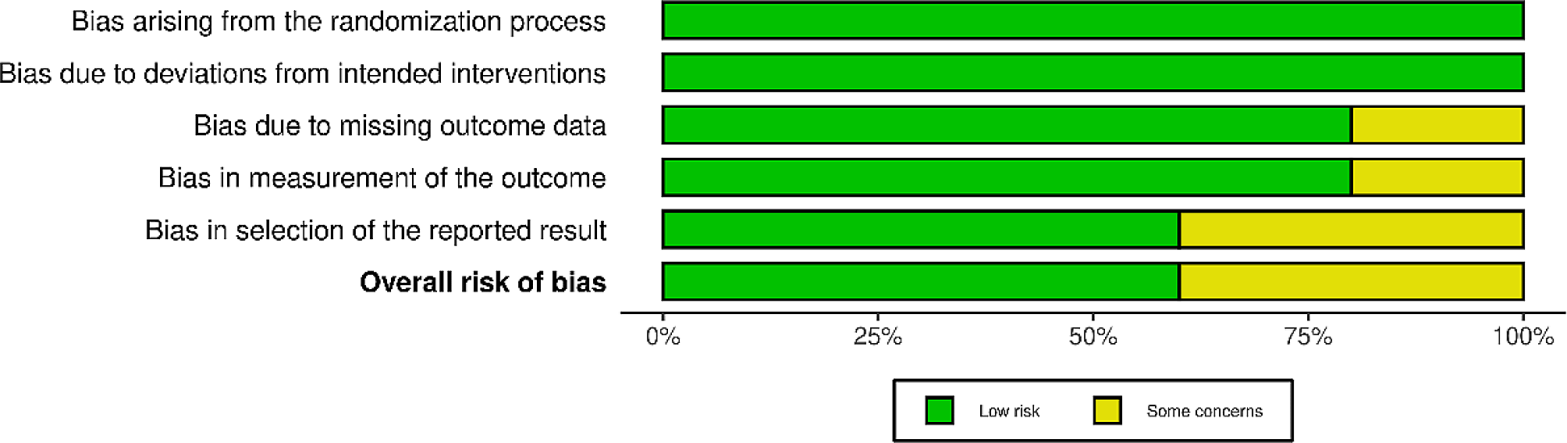
Summary of the quality assessment of the included studies

We assessed the quality of the five selected randomized controlled trials using the ROB-2 criteria, finding that three of them had a low risk of bias [6, 9, 12], while the remaining two raised some concerns score [5, 11] in terms of bias (Figs. 2 and 3).

### Synthesis of results

Overall, five studies with total sample size of 302 participants for both Dexmedetomidine and Remifentanil included for meta-analysis.

#### Heart rate (HR) and mean arterial pressure (MAP) during surgery

**Fig. 4:**
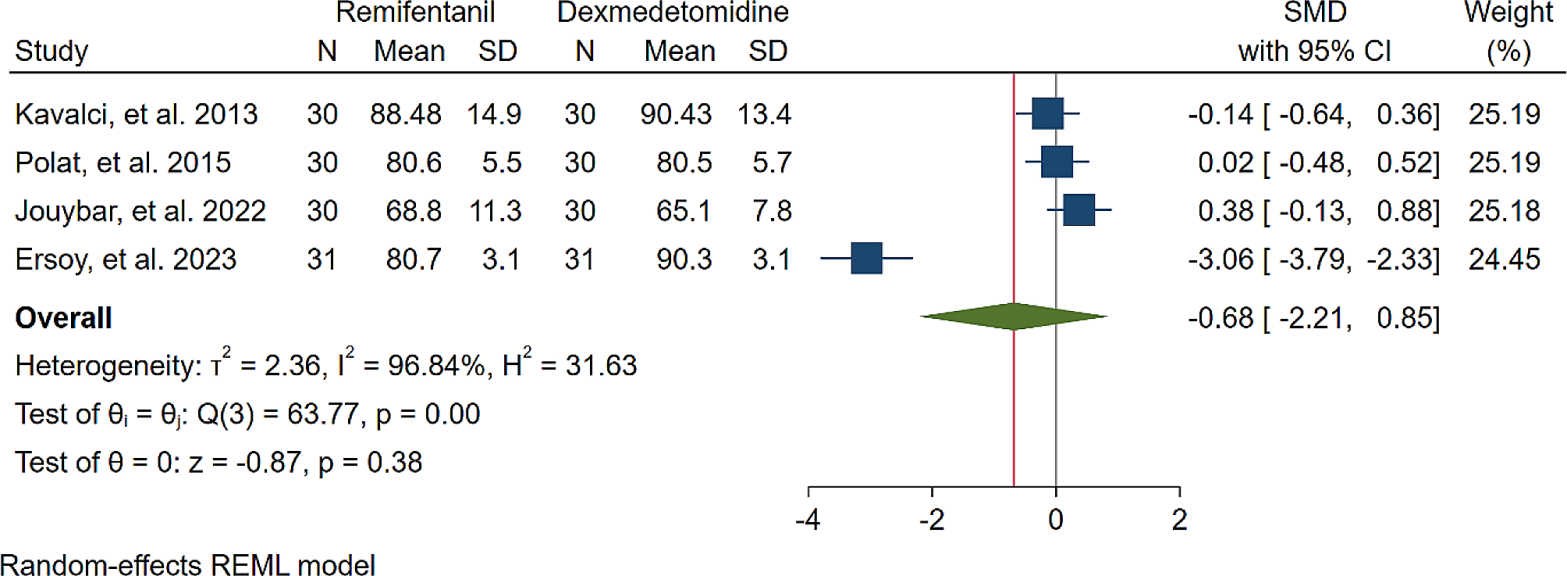
Comparative Meta-analysis of Heart Rate Variation Between Dexmedetomidine and Remifentanil During Intubation

**Fig. 5:**
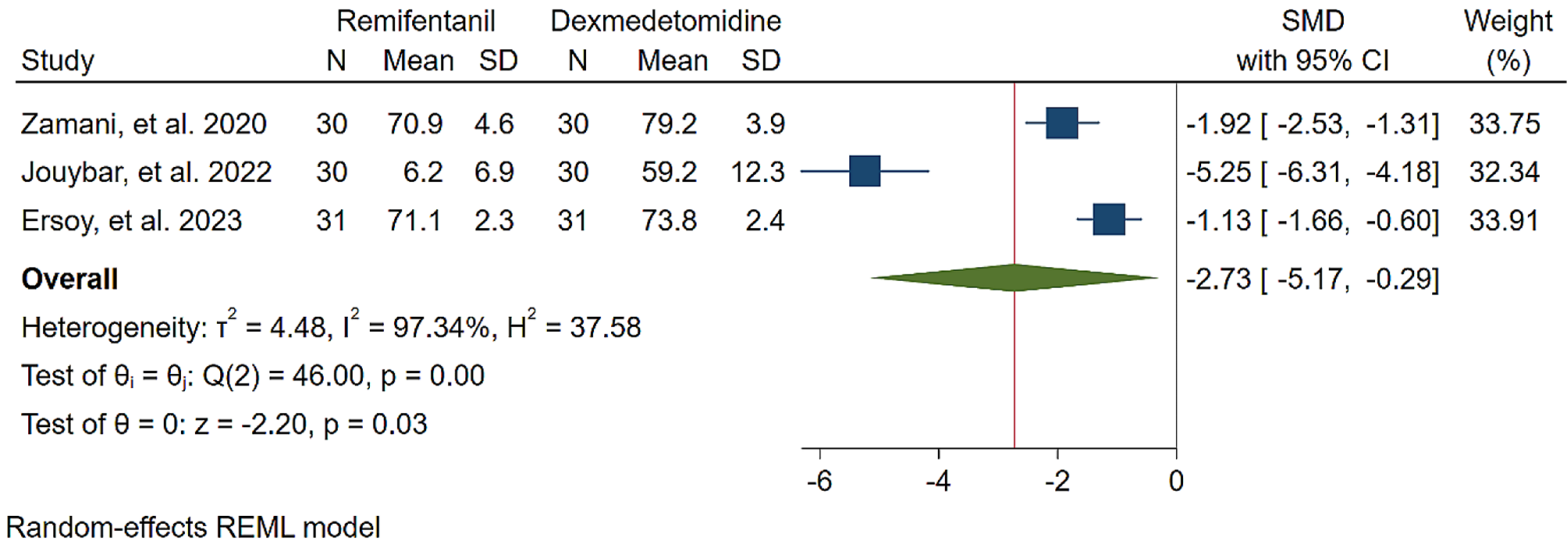
Comparative Meta-analysis of Heart Rate Variation Between Dexmedetomidine and Remifentanil at 15th minute of surgery

**Fig. 6:**
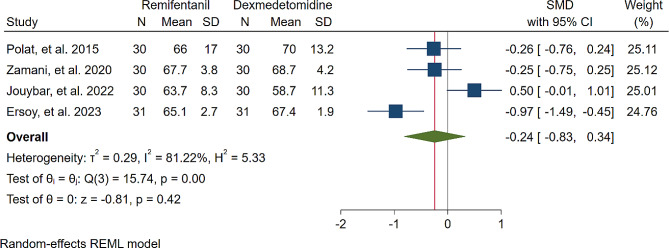
Comparative Meta-analysis of Heart Rate Variation Between Dexmedetomidine and Remifentanil at 30th minute of surgery

**Fig. 7:**
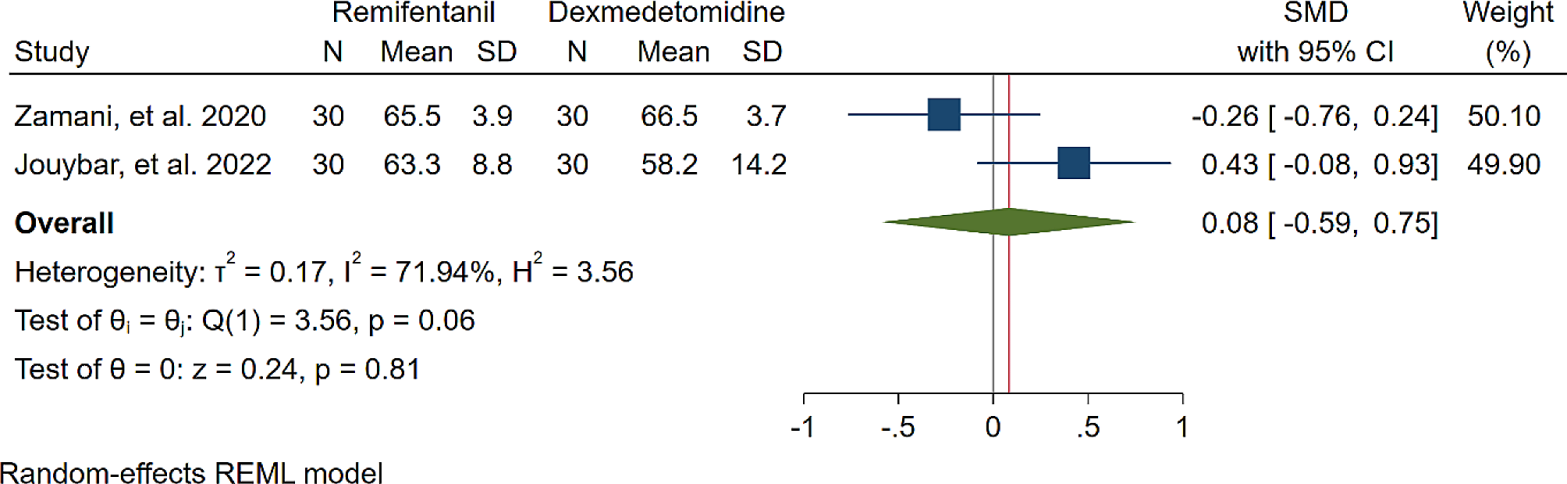
Comparative Meta-analysis of Heart Rate Variation Between Dexmedetomidine and Remifentanil at 45th minute of surgery

**Fig. 8:**
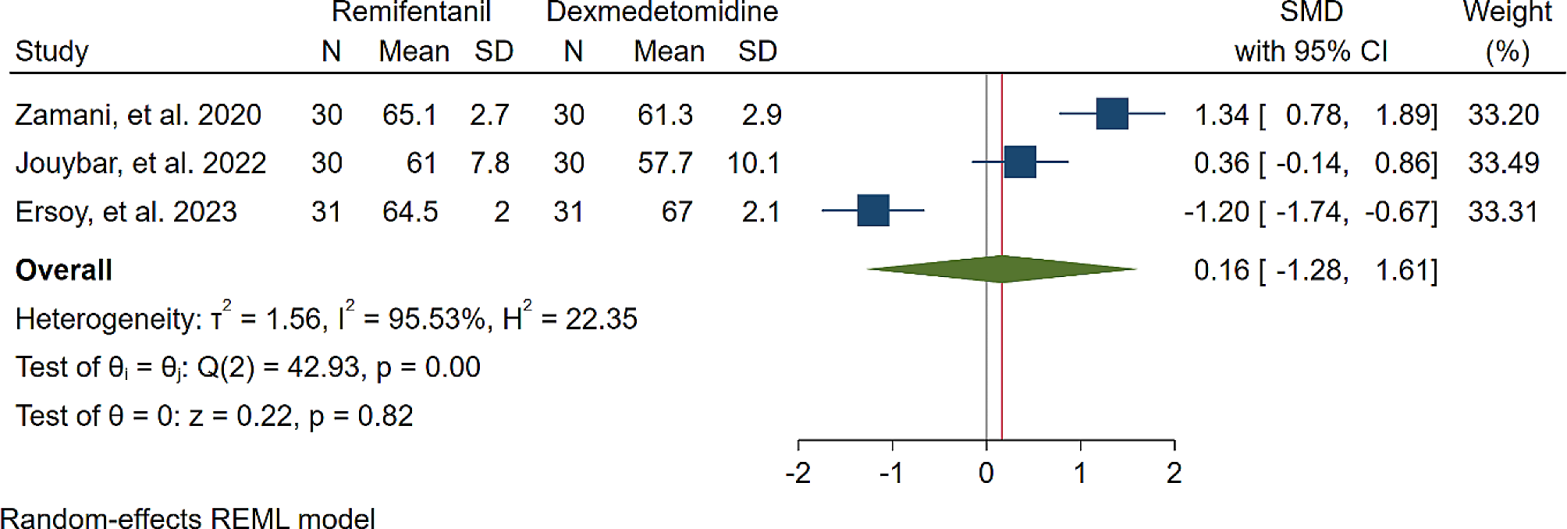
Comparative Meta-analysis of Heart Rate Variation Between Dexmedetomidine and Remifentanil at 60th minute of surgery

**Fig. 9:**
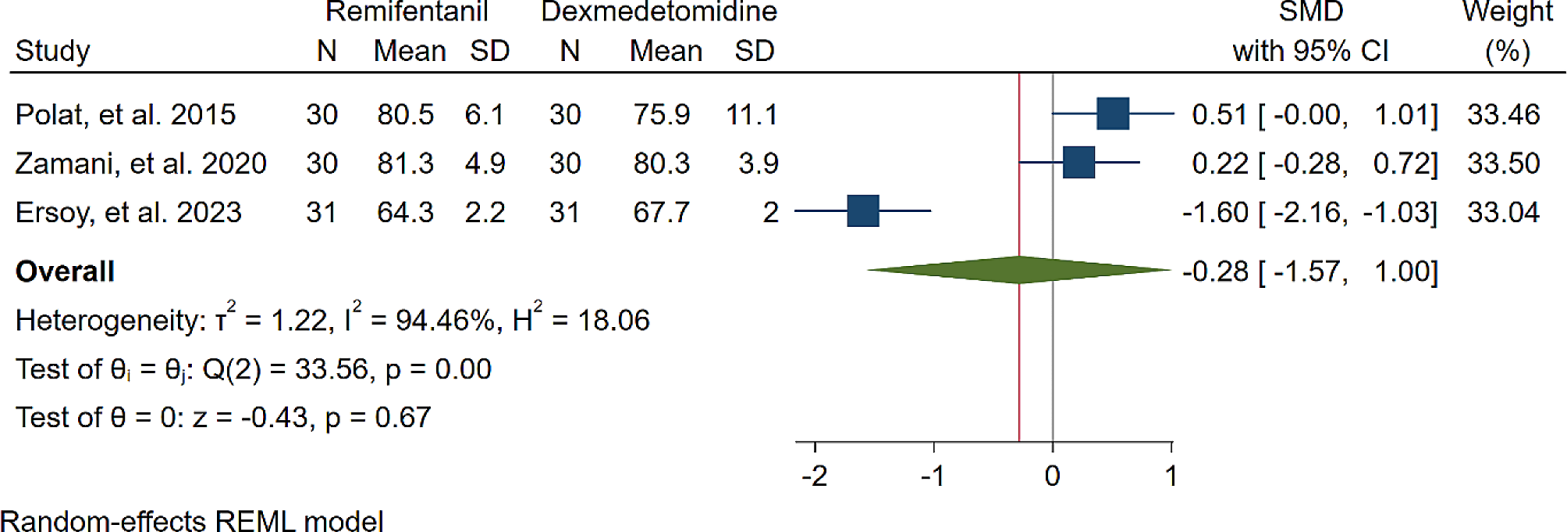
Comparative Meta-analysis of Heart Rate Variation Between Dexmedetomidine and Remifentanil During extubation

**Fig. 10:**
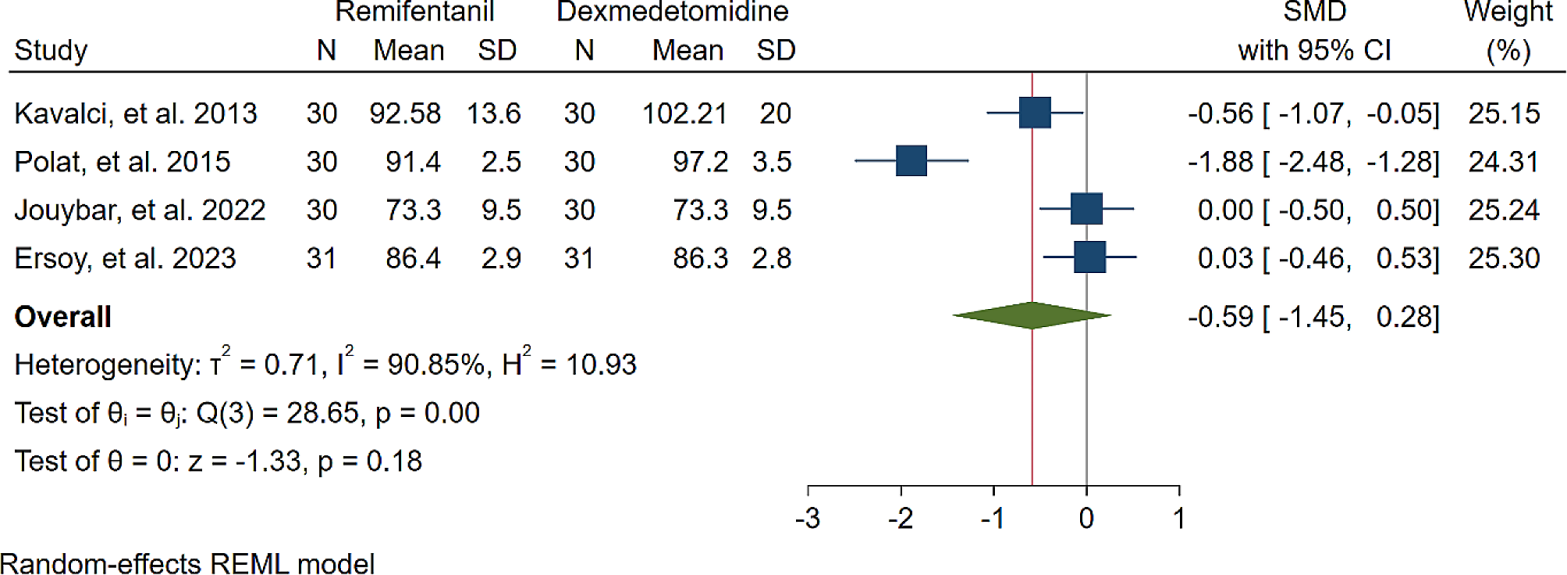
Comparative Meta-analysis of Mean Arterial Pressure Variation Between Dexmedetomidine and Remifentanil During Intubation

**Fig. 11:**
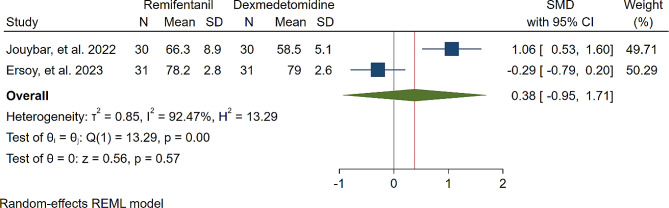
Comparative Meta-analysis of Mean Arterial Pressure Variation Between Dexmedetomidine and Remifentanil at 15th minute of surgery

**Fig. 12:**
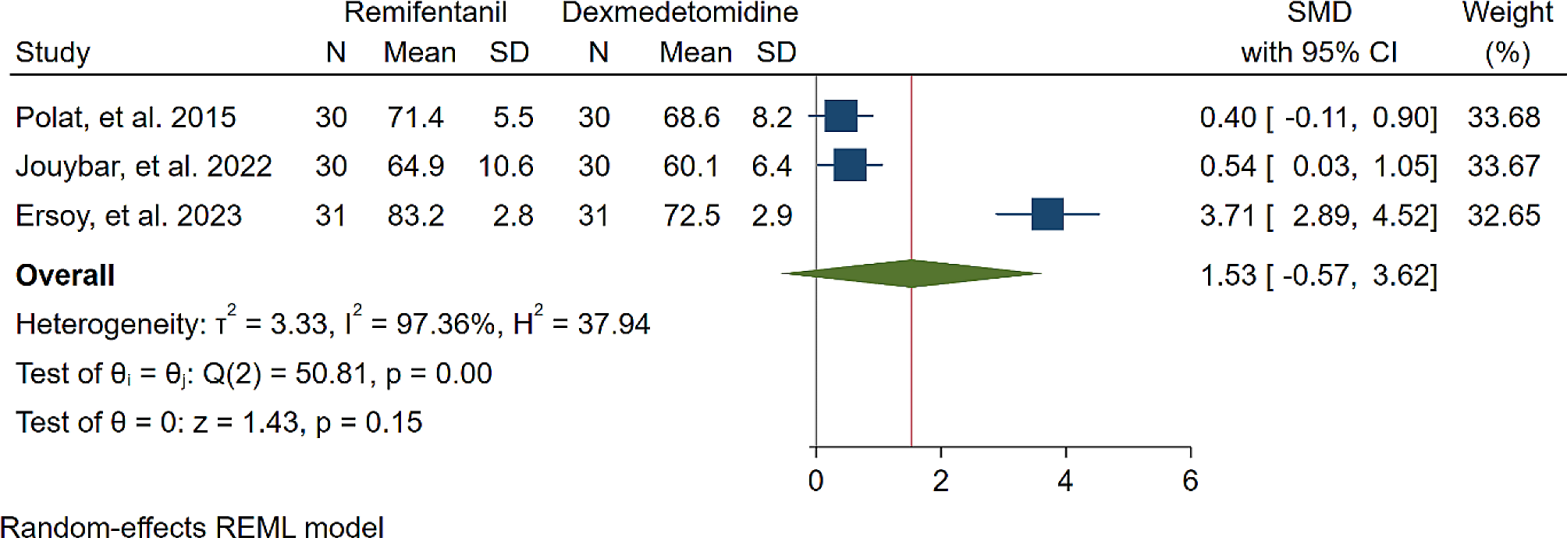
Comparative Meta-analysis of Mean Arterial Pressure Variation Between Dexmedetomidine and Remifentanil at 30th minute of surgery

**Fig. 13:**
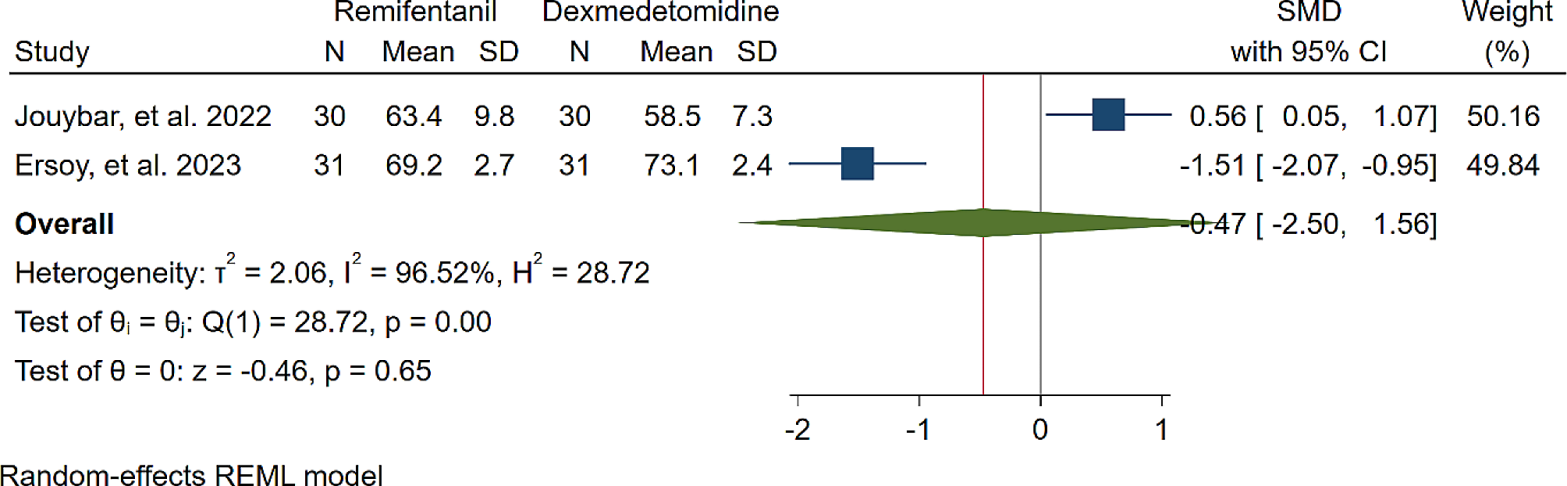
Comparative Meta-analysis of Mean Arterial Pressure Variation Between Dexmedetomidine and Remifentanil at 60th minute of surgery

**Fig. 14:**
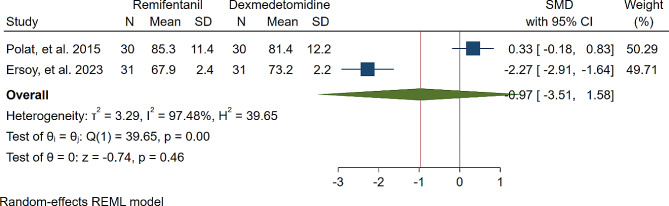
Comparative Meta-analysis of Mean Arterial Pressure Variation Between Dexmedetomidine and Remifentanil During extubation

A study was conducted to assess the effects of Dexmedetomidine and Remifentanil on HR and MAP during surgery. The evaluation included monitoring HR and MAP at various time intervals: during intubation, and at the 15th, 30th, 45th, and 60th minutes of surgery, as well as during extubation. The findings indicated that there were no significant variations in MAP and HR between the Dexmedetomidine and Remifentanil groups, except for a lower HR observed in the Remifentanil group at the 15th minute of surgery (Standardized Mean Difference: -0.24 [-0.83, 0.34]; I2: 81.22%) (Table 3) (Figs. 4, 5, 6, 7, 8, 9, 10, 11, 12, 13 and 14).

**Table 3:** A meta-analysis comparing the impact of Dexmedetomidine and Remifentanil on heart rate and mean arterial pressure

Variable	HR	SMD	I^2^ (%)	MAP	SMD	I^2^ (%)
Time of measurement	Number of studies/participants			Number of studies/participants		
After intubation	4/242	-0.68 [-2.21, 0.85]	96.84	4/242	-0.59 [-1.45, 0.28]	90.85
15th min	3/182	-2.73 [-5.17, -0.29]*	97.34	2/122	0.35 [-0.95, 1.71]	92.47
30th min	4/242	-0.24 [-0.83, 0.34]	81.22	3/182	1.53 [-0.57, 3.62]	97.36
45th min	2/120	0.08 [-0.59, 0.75]	71.94	NA	NA	NA
60th min	3/182	0.16 [-1.28, 1.61]	95.53	2/122	-0.47 [-2.50, 1.56]	96.52
After extubation	3/182	-0.28 [-1.57, 1.00]	94.46	2/122	-0.97 [-3.51, 1.58]	97.48

*Significant

#### Outcomes after surgery

To evaluate the impact of Dexmedetomidine and Remifentanil on post-surgery results, various factors were examined, including pain levels (measured at extubating, 15 min after surgery, and 30 min after surgery), the requirement for pain relief medication, satisfaction levels of both patients and surgeons, agitation scores (at the 15th and 30th minute after surgery), and recovery time. The analysis revealed no substantial distinctions between Dexmedetomidine and Remifentanil in any of these aspects (Supplementary Material 2, Figure S1-9).

## Discussion

In this meta-analysis, our objective was to compare the outcomes of nasal surgery, such as heart rate, blood pressure, satisfaction, pain, agitation, and recovery time, among patients sedated using dexmedetomidine and remifentanil. Following a thorough review of five pertinent clinical trials comparing the efficacy of remifentanil and dexmedetomidine for sedation during rhinoplasty, no significant differences were found in intraoperative MAP, HR, bleeding, recovery time, postoperative pain scores, surgeon and patient satisfaction, and agitation scores. This analysis specifically pertains to patients undergoing rhinoplasty.

Rhinoplasty is a prevalent cosmetic surgery globally [17]. The anesthesia administered involves three categories: anxiolytics like benzodiazepines, narcotics such as short-acting fentanyl, and sedative antiemetics like diphenhydramine [18, 19].

Remifentanil is a potent ultra-short-acting analgesic that exhibits a strong affinity for opioid receptors. It effectively mitigates the stress induced by tracheal intubation and surgical manipulation during the maintenance of general anesthesia [20, 21]. This derivative of fentanyl reduces blood pressure by triggering the release of histamine and centrally deactivating vasoconstriction [22]. In comparison to other opioids, it enhances hemodynamic stability and maintains cerebral blood flow during demanding surgical procedures [23]. Remifentanil administered via patient-controlled analgesia has shown inhibitory effects on apoptotic pathways and oxidative stress, thereby providing organ protection and reducing inflammatory responses [24–26]. Kosucu and colleagues conducted a double-blinded clinical trial to evaluate the impact of remifentanil with controlled hypotension on intraoperative bleeding, postoperative edema, and ecchymosis in rhinoplasty patients. The study demonstrated that the opioid derivative led to a decrease in MAP during surgery, resulting in reduced bleeding [27]. The authors found that utilizing remifentanil with controlled hypotension led to decreased postoperative swelling and bruising of the eyelids. Dexmedetomidine, acting as an alpha agonist, possesses anxiolytic, sedative, and analgesic properties without any reported side effects [28]. By blocking alpha receptors in the brainstem and inhibiting norepinephrine release, this sedative medication has shown potential in reducing bleeding, as well as enhancing patient satisfaction and safety [29]. Several studies have underscored the significant impact of dexmedetomidine in reducing bleeding during septorhinoplasty [30–32]. Rokh et al. demonstrated that long-acting dexmedetomidine notably reduces intraoperative bleeding compared to magnesium sulfate in patients undergoing rhinoplasty [33]. In a study comparing tranexamic acid and dexmedetomidine for controlling bleeding rates during procedures, despite their similar efficacy, dexmedetomidine was favored as a better option for rhinoplasty due to lower bleeding levels, while tranexamic acid was found to be more effective in major surgeries [34]. The study found no significant difference in bleeding amounts between dexmedetomidine and remifentanil. HR and MAP during surgery exhibited no significant variations, with MAP and HR being crucial factors influencing surgical quality and outcomes. Maintaining lower blood pressure is linked to reduced blood loss and a clearer surgical field. Controlled hypotension, commonly defined as a 30% decrease in MAP, may vary due to reported cases of organ failure, including acute kidney injury, myocardial injury, and mortality during the procedure [35–38]. Zamani et al. found that while both sedatives decreased bleeding during surgery, patients who received remifentanil experienced more pronounced outcomes [11]. Polat and colleagues noted comparable hemodynamic statuses between groups, consistent with the findings of Kavalci et al. and Ersoy et al. [5, 9, 12]. Another clinical trial reported a lower incidence of bleeding in patients who received dexmedetomidine compared to those who received remifentanil [6]. The studies mentioned exhibit notable biases that may significantly impact their outcomes. Issues such as a restricted number of patients, selection bias, modified anesthetic protocols, inattentive hemodynamic recording, and the absence of a placebo require thorough assessment and standardization in future controlled studies. From a specific perspective, dexmedetomidine is associated with common adverse events such as rhinorrhea, severe hypotension, hypertension, and notably, bradycardia [39, 40]. Motlagh and colleagues suggested using lower loading doses of the alpha agonist to reduce alpha stimulation of vascular smooth muscles. This approach aims to maintain optimal hypotension, ensure a clear field, and prevent life-threatening bradycardia by preserving central sympathetic outflow [41]. Rashad et al. confirmed that dexmedetomidine not only maintains preferred hemodynamics but also reduces the need for postoperative analgesics and decreases the occurrence of nausea and vomiting [42]. Remifentanil exerts protective effects on ischemic reperfusion organ injuries by reducing intracellular calcium, activating anti-apoptotic pathways, and regulating reactive oxygen species, as viewed from another perspective [43–45]. The analysis indicated lower hazard ratios (HRs) only within the initial 15 min following remifentanil induction (SMD: -0.24 [-0.83, 0.34]; I2: 81.22%). This variation might be linked to differing HRs at different stages of rhinoplasty due to pain, induced stress, and individual surgical approaches. Surgeons’ unique methods could also impact patients’ HR. Moreover, intraoperative cardiac outcomes are significantly influenced by factors such as preoperative preparedness, stress regarding post-surgery pain, revision surgery, aesthetic concerns, and anxiety about ongoing nasal obstruction [46].

Jouybar et al. discovered that patients administered dexmedetomidine during anesthesia experienced significantly greater surgical satisfaction, attributed to a reduced amount of bleeding [6]. Another study found that using dexmedetomidine instead of midazolam alongside morphine and propofol resulted in shorter surgery times, lower pain scores, and increased patient and surgical satisfaction [47]. However, this study found no significant difference in surgical satisfaction and the quality of the surgical field. It’s important to note that the quality of the surgical field varied and was not consistently reported in the included studies. Ersoy and colleagues acknowledged surgical field bias as a significant limitation in their study [5]. In a recent double-blinded clinical trial comparing the use of remifentanil sedation with isoflurane versus propofol, researchers found that the combination of isoflurane-remifentanil led to more favorable outcomes, including controlled hypotension and increased surgical satisfaction [48]. To prevent further controversies, future research should focus on assessing the impact of maintenance and induction dosages of dexmedetomidine and remifentanil in terms of their combined sedative effects.

Earlier research findings indicated increased satisfaction with surgery, stable hemodynamics, and reduced intraoperative blood loss when using the alpha agonist [22, 49]. The authors observed increased patient satisfaction and reduced post-surgical pain when using fentanyl derivatives. However, our findings contradicted this, showing no significant difference in pain levels between dexmedetomidine and remifentanil during extubation, 15 min, or 30 min after surgery, as well as in postoperative analgesic uptake. Other studies reported lower Visual Analog Scale (VAS) scores for remifentanil patients in terms of initial pain relief [6]. Additionally, Polat and colleagues, confirming Ersoy et al.‘s results, demonstrated lower pain scores in patients who received Dexmedetomidine [5, 12]. In addition to the limitations mentioned in the included studies, further reports have addressed post-rhinoplasty pain. Throat pain may occur in some patients due to intubation, with no statistically significant differences observed among groups with various anesthesia methods [50]. Pain and vas scores are subjective measurements influenced by patients’ characteristics. Women with menstrual pains exhibit both increased resistance and vulnerability to pain compared to men [51]. A study examining pain intensity following rhinoplasty found no statistically significant difference between genders [52]. Another study emphasized the significance of confidence in influencing the perception of pain [53]. After rhinoplasty, a study found no correlation between cosmetic satisfaction, confidence, and the perception of pain [52]. After rhinoplasty, pain location was categorized into seven areas for various patients. These areas include the tip of the nose, nasal bone, inside of the nose, upper teeth and lips, nasal wings, eyes, and forehead [54]. Changes in neural conduction may impact how patients describe their pain. Pain plays a crucial role in determining patient satisfaction. Gadkaree et al. demonstrated that patients with lower-than-expected pain intensity tended to show greater functional improvement and, as a result, higher satisfaction [52]. A survey of 2326 patients who underwent rhinoplasty revealed that females exhibited notably higher satisfaction levels compared to males [55]. The main dissatisfactions were primarily related to a remaining dorsal hump and under-rotated tip, with similar concerns expressed by both men and women. In men, the third most common issue was a nose that was deemed too small, while in women, it was a bulbous tip [55]. Patient satisfaction rates were found to be correlated with preoperative body appreciation and nasal obstructions [56]. In summary, information on subjective and objective pain variables, patients’ prior pain perceptions, analgesic intake, and the use of opioids or intranasal graft types like batten, caudal, or spreader may influence postoperative pain and narcotic requirements. Additional assessments focusing on classifying rhinoplasty cases and controlling other pain-related factors could enhance the understanding of how anesthetic medications impact patient satisfaction and pain perception.

Agitation, often observed in the initial stages of recovery following rhinoplasty, is characterized by feelings of confusion, tears, and restlessness [57]. A prior investigation indicated that administering ketamine at sub-analgesic doses can avert this prevalent complication [58]. A meta-analysis emphasized reduced surgical duration, bleeding, and opioid inhalation with systemic administration of dexmedetomidine compared to a placebo [59]. The authors also noted a decrease in postoperative pain and emergence agitation. However, there was no discernible difference in agitation scores between the two groups, aligning with the findings of Kavalci and colleagues [9]. Jouybar et al. showed reduced agitation scores with remifentanil, as opposed to higher scores [6]. Several potential risk factors for emergence agitation after surgery include age, sex, obesity, ethnicity, intubation attempts, type of surgery, duration, and pre-existing psychiatric or cognitive problems [60–64]. Chronic lung disease, smoking and drinking history, postoperative pain intensity, nausea and vomiting, and the use of a urine catheter, chest, or tracheal tube were identified as relevant risk factors [61, 62, 64]. In comparison to saline, Polat and colleagues discovered that without loading, the maintenance dose of both anesthetics is more effective in preventing emergence agitation. Additionally, dexmedetomidine resulted in lower vomiting [12]. The studies did not report the blood concentration of carbon dioxide. Both low carbon dioxide levels, leading to reduced cerebral blood flow, and acidosis induced by high carbon dioxide levels contributed to the emergence of agitation [65].

Our study revealed no significant difference in the post- and intra-operative outcomes of rhinoplasty when comparing the effects of alpha agonist and derivative opioid analgesics. This meta-analysis, the first of its kind for these recently used anesthetics, is subject to limitations, including a small sample size (5 trials and 302 participants), which weakens the reliability and evidence supporting the comparison of dexmedetomidine and remifentanil. Additionally, varying induction doses in the included articles, as noted by Motlagh et al. [41], may lead to complications such as uncontrolled bradycardia, hypotension, or hypertension. The study emphasizes the significance of distinct protocols for the induction and maintenance of sedation for remifentanil and dexmedetomidine due to their varied half-life and peak effects. It recommends future research to establish standardized dosage protocols. Additionally, it notes the absence of a control group comparison or placebo in the included study, variations in scales for agitation, pain, and satisfaction across studies, and the influence of multiple factors on postoperative assessments. The need for future controlled trials to evaluate the efficacy of each anesthetic, considering both surgical and non-surgical factors, is underscored.

### Conclusion

This study marks the first meta-analysis of clinical trials investigating the effects of dexmedetomidine and remifentanil on post- and intra-operative outcomes in nasal surgeries. The analysis found no significant differences between the two groups. Surgical outcomes following sedation with remifentanil and dexmedetomidine showed no discernible distinctions in any aspects of nasal surgeries. However, it underscores the importance of considering the potential impact of varied sedation methods and medications. The findings suggest a necessity for further evaluations to establish a standardized and more effective anesthetic approach, with the goal of reducing surgical and anesthesia-related complications while optimizing surgical outcomes.

### Electronic supplementary material

Below is the link to the electronic supplementary material.

Supplementary Material 1

Supplementary Material 2

## Data Availability

All information required is given in the text and supplementary materials, other supplementary information can be obtained upon email from the corresponding author.
