# Supplementary material for: Dexmedetomidine versus remifentanil in nasal surgery: a systematic review and meta-analysis
Source: BMC Anesthesiol. 2024 May 30;24:194. doi: 10.1186/s12871-024-02563-0 (PMC11138079; doi:10.1186/s12871-024-02563-0)
Supplement: Supplementary file 1 — Supplementary Material 1 [file 12871_2024_2563_MOESM1_ESM.docx]

**Databases**

1. PubMed: 9 results

#1: ((((((((((((("Rhinoplasty"[Mesh]) OR (Rhinoplasty[Title/Abstract])) OR ("Nasal reconstruction"[Title/Abstract])) OR (Rhinoplast*[Title/Abstract])) OR ("Nose reconstruction"[Title/Abstract])) OR ("nose surgery"[Title/Abstract])) OR (septorhinoplasty[Title/Abstract])) OR ("nose job"[Title/Abstract])) OR (septoplasty[Title/Abstract])) OR ("revisional rhinoplasty"[Title/Abstract])) OR ("augmentation rhinoplasty"[Title/Abstract])) OR (Rhinoplasties[Title/Abstract])) OR ("Nasal Surgery"[Title/Abstract])) OR ("Nasal Surgical Procedure"[Title/Abstract])

#2: (Remifentanil*[Title/Abstract]) OR ("Remifentanil"[Mesh])

#3: ((Dexmedetomidine*[Title/Abstract]) OR (Precedex[Title/Abstract])) OR ("Dexmedetomidine"[Mesh])

Final: #1 AND #2 AND #3

1. Scopus: 25 results

#1: TITLE-ABS-KEY(Rhinoplasty) OR TITLE-ABS-KEY(“Nasal reconstruction”) OR TITLE-ABS-KEY(Rhinoplast*) OR TITLE-ABS-KEY(“Nose reconstruction”) OR TITLE-ABS-KEY(“nose surgery”) OR TITLE-ABS-KEY(septorhinoplasty) OR TITLE-ABS-KEY(“nose job”) OR TITLE-ABS-KEY(septoplasty) OR TITLE-ABS-KEY(“revisional rhinoplasty”) OR TITLE-ABS-KEY(“augmentation rhinoplasty”) OR TITLE-ABS-KEY(Rhinoplasties) OR TITLE-ABS-KEY(“Nasal Surgery”) OR TITLE-ABS-KEY(“Nasal Surgical Procedure”)

#2: TITLE-ABS-KEY(Remifentanil*)

#3: TITLE-ABS-KEY(Dexmedetomidine*) OR TITLE-ABS-KEY(Precedex)

Final: #1 AND #2 AND #3

1. WOS: 15 results

#1: TS=(Rhinoplasty OR “Nasal reconstruction” OR Rhinoplast* OR “Nose reconstruction” OR “nose surgery” OR septorhinoplasty OR “nose job” OR septoplasty OR “revisional rhinoplasty” OR “augmentation rhinoplasty” OR Rhinoplasties OR “Nasal Surgery” OR “Nasal Surgical Procedure”)

#2: TS=(Remifentanil*)

#3: TS=(Dexmedetomidine* OR Precedex)

Final: #1 AND #2 AND #3

1. Cochrane central: 14 results

#1: (Rhinoplasty):ti,ab,kw OR (“Nasal reconstruction”):ti,ab,kw OR (Rhinoplast*):ti,ab,kw OR (“Nose reconstruction”):ti,ab,kw OR (“nose surgery”):ti,ab,kw OR (septorhinoplasty):ti,ab,kw OR (“nose job”):ti,ab,kw OR (septoplasty):ti,ab,kw OR (“revisional rhinoplasty”):ti,ab,kw OR (“augmentation rhinoplasty” ):ti,ab,kw OR (Rhinoplasties):ti,ab,kw OR (“Nasal Surgery” ):ti,ab,kw OR (“Nasal Surgical Procedure”)

#2: (Remifentanil):ti,ab,kw

#3: (Dexmedetomidine*):ti,ab,kw OR (Precedex):ti,ab,kw

Final: #1 AND #2 AND #3

Google scholar: 7 results

allintitle: Remifentanil Dexmedetomidine Rhinoplasty OR septorhinoplasty OR "Nasal Surgery"
