# Supplementary material for: Dexmedetomidine versus remifentanil in nasal surgery: a systematic review and meta-analysis
Source: BMC Anesthesiol. 2024 May 30;24:194. doi: 10.1186/s12871-024-02563-0 (PMC11138079; doi:10.1186/s12871-024-02563-0)
Supplement: Supplementary file 2 — Supplementary Material 2 [file 12871_2024_2563_MOESM2_ESM.docx]

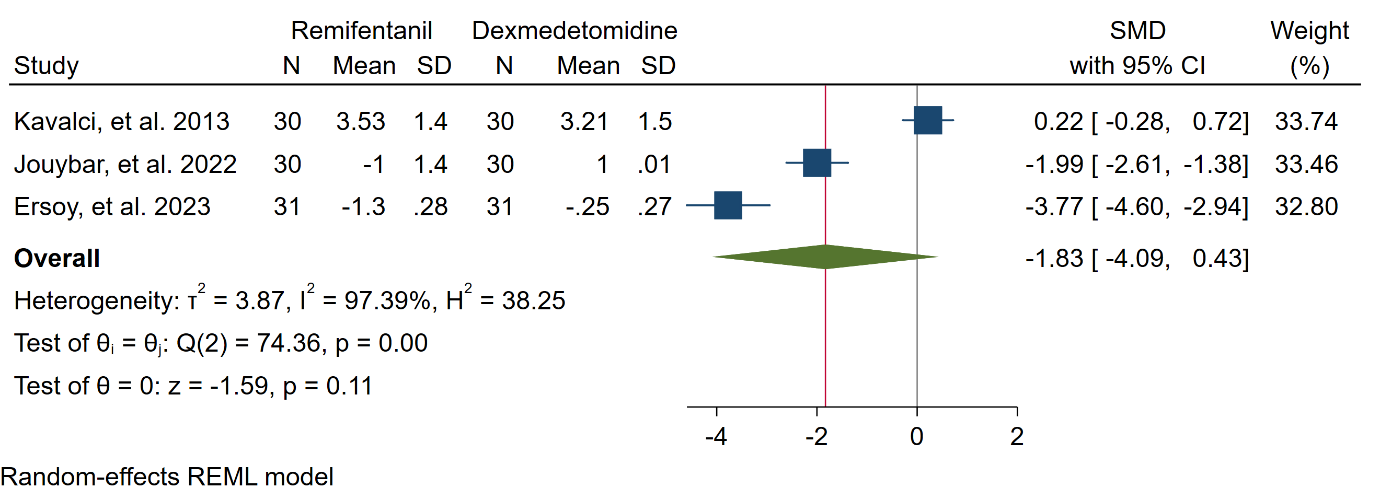


Figure S1. Comparative Meta-analysis of agitation score Variation Between Dexmedetomidine and Remifentanil During extubation


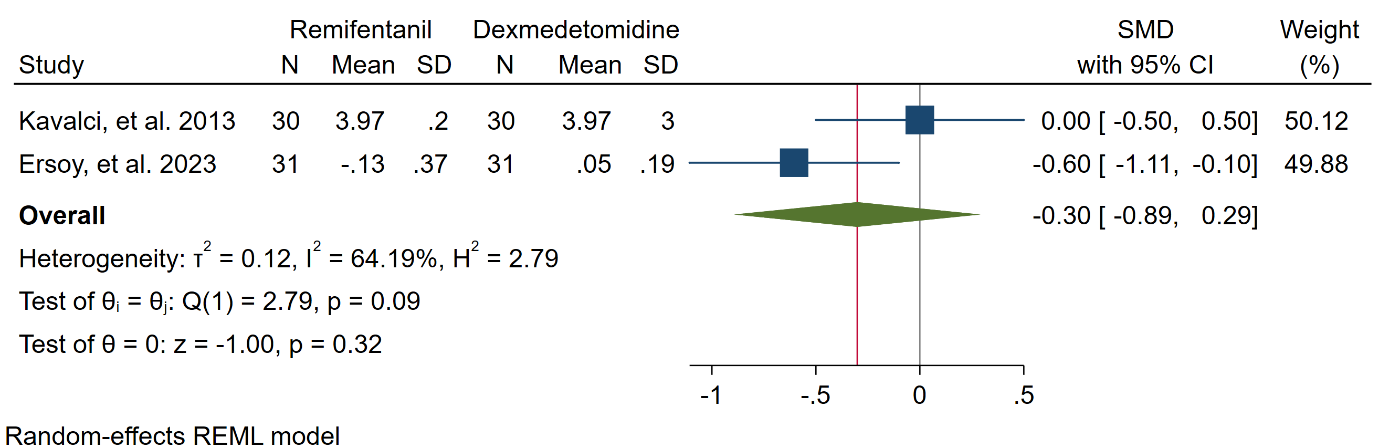


Figure S2. Comparative Meta-analysis of agitation score Variation Between Dexmedetomidine and Remifentanil at 30^th^ minute after extubation


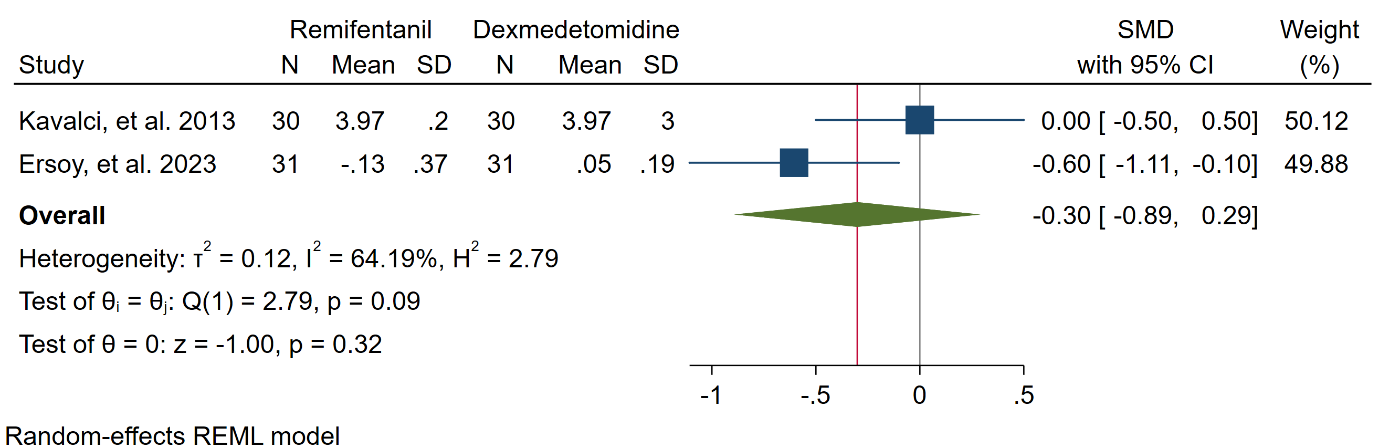


Figure S3. Comparative Meta-analysis of need to analgesics Variation Between Dexmedetomidine and Remifentanil after extubation


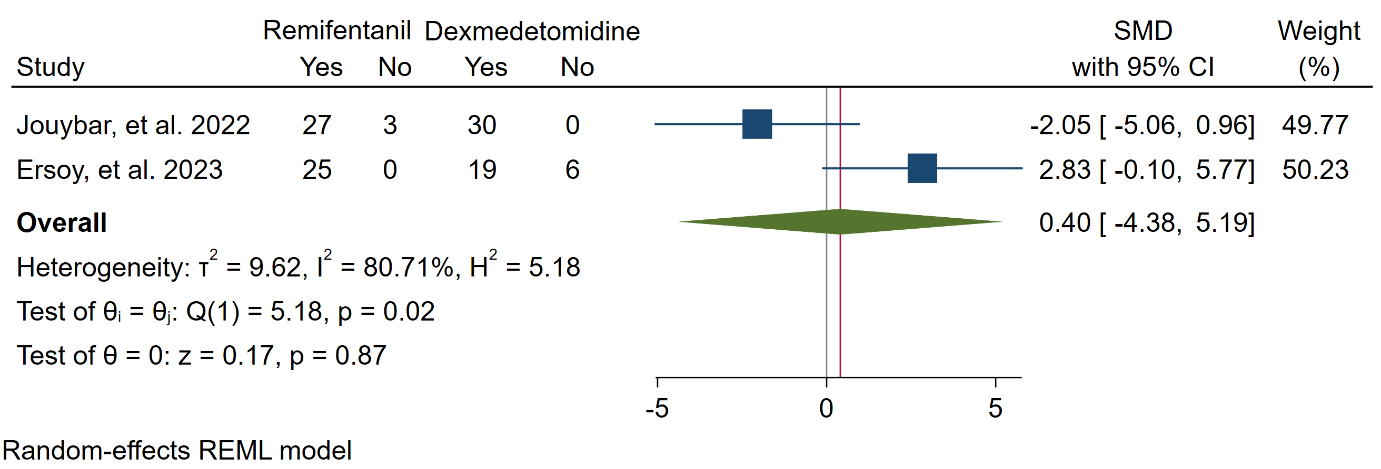


Figure S4. Comparative Meta-analysis of surgeon satisfaction Variation Between Dexmedetomidine and Remifentanil


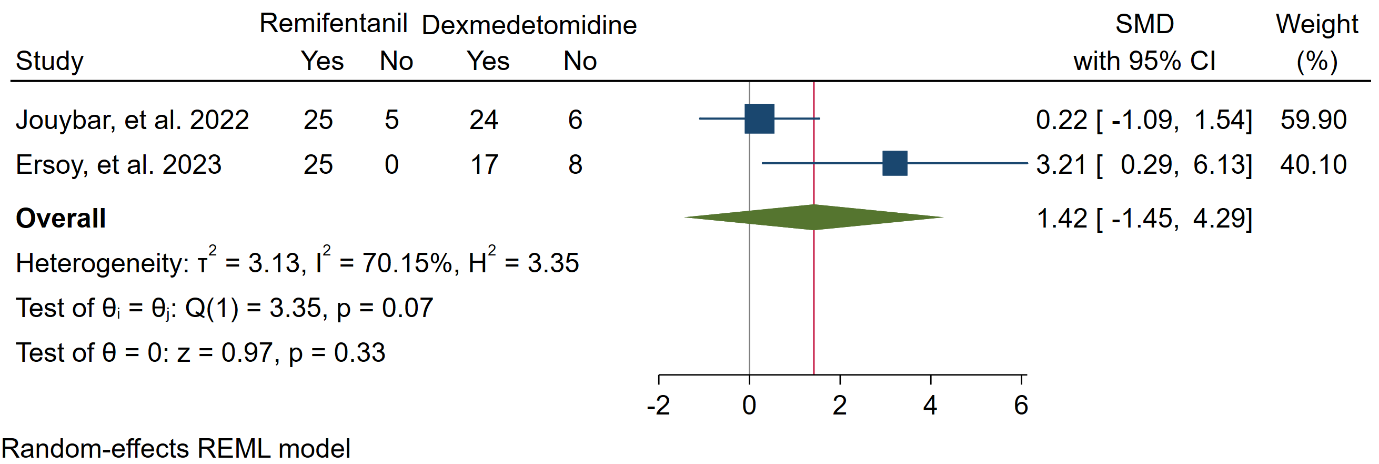


Figure S5. Comparative Meta-analysis of patient satisfaction Variation Between Dexmedetomidine and Remifentanil


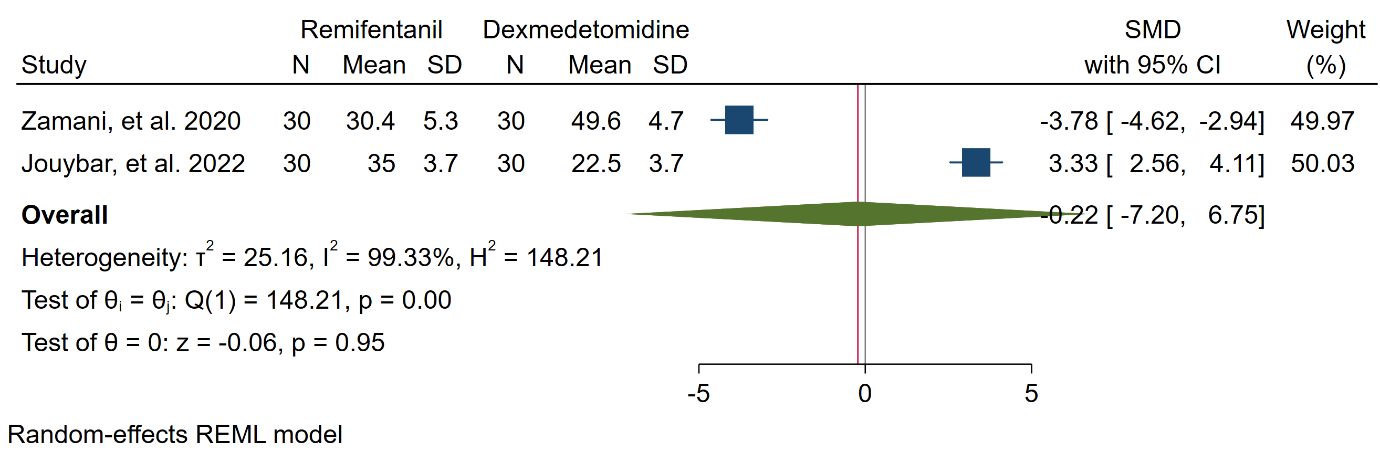


Figure S6. Comparative Meta-analysis of recovery time Variation Between Dexmedetomidine and Remifentanil


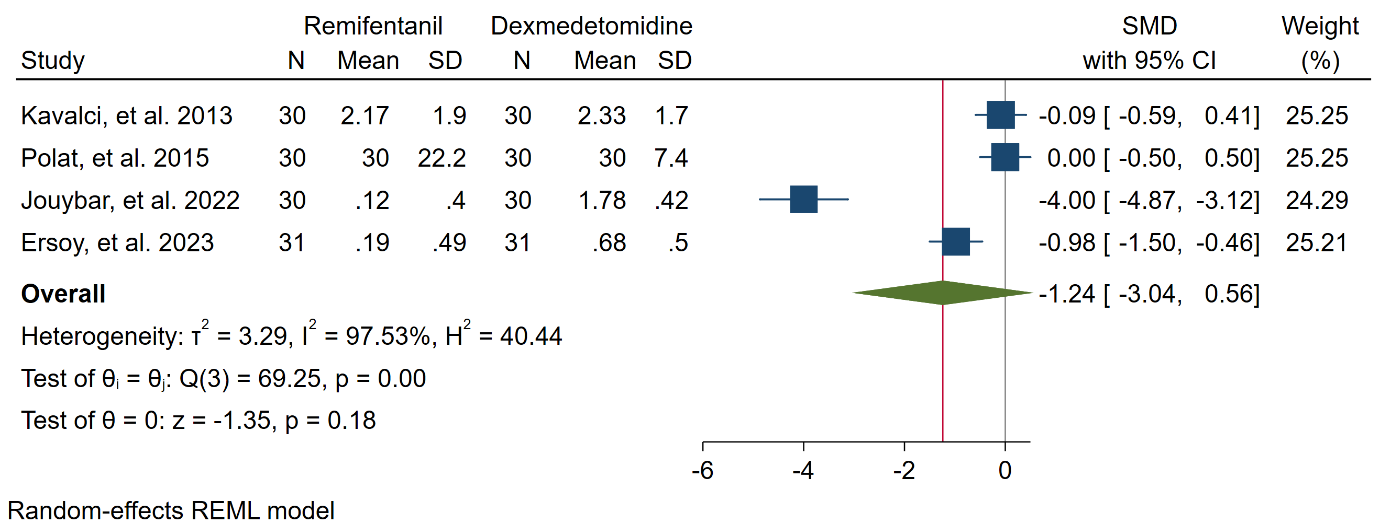


Figure S7. Comparative Meta-analysis of pain score Variation Between Dexmedetomidine and Remifentanil during extubation


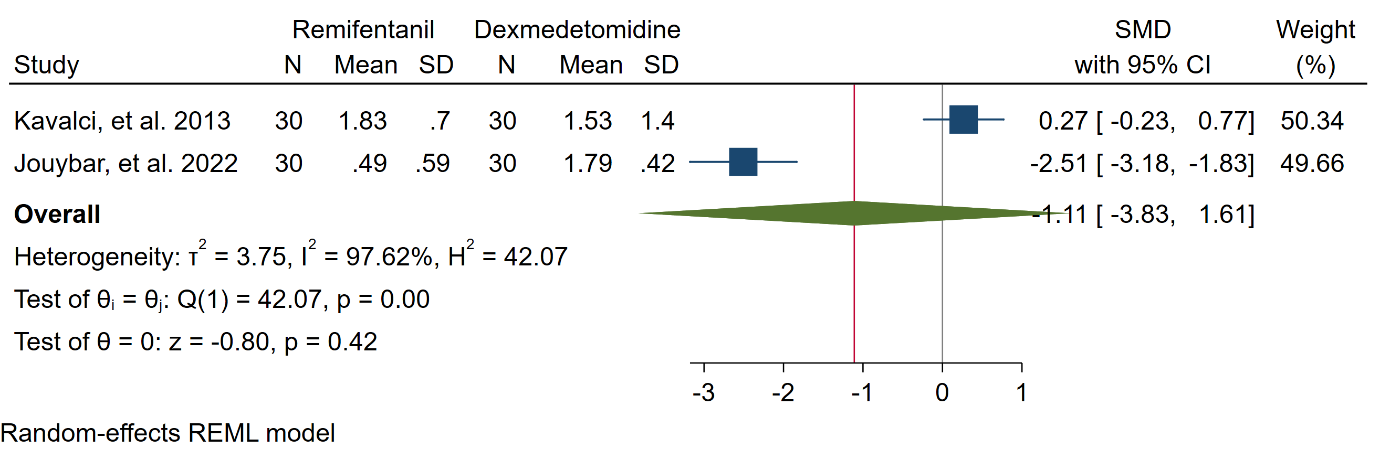


Figure S8. Comparative Meta-analysis of pain score Variation Between Dexmedetomidine and Remifentanil at 15^th^ minute after extubation


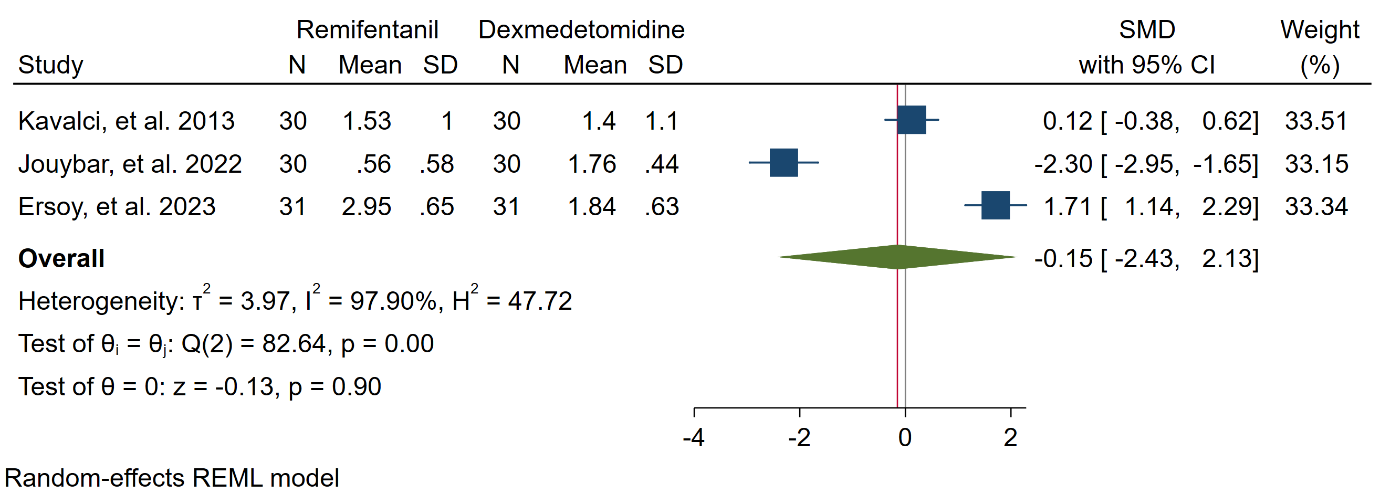


Figure S9. Comparative Meta-analysis of pain score Variation Between Dexmedetomidine and Remifentanil at 30^th^ minute after extubation
